# ﻿*Craspedostaurosnazmii* sp. nov., a new diatom species (Bacillariophyta) from the Turkish Coast of the Black Sea

**DOI:** 10.3897/phytokeys.232.106545

**Published:** 2023-09-11

**Authors:** Elif Yılmaz, Andrzej Witkowski, Neslihan Özdelice, Cüneyt Nadir Solak, Romain Gastineau, Turgay Durmuş

**Affiliations:** 1 Institute of Marine and Environmental Sciences, University of Szczecin, Mickiewicza 16A, Szczecin, PL70–383 Poland Kütahya Dumlupınar University Kütahya Turkiye; 2 Department of Biology, Faculty of Science and Art, Kütahya Dumlupınar University, 43000 Kütahya, Turkiye University of Szczecin Szczecin Poland; 3 Istanbul University, Faculty of Science, Department of Biology, 34134 Istanbul, Turkiye Istanbul University Istanbul Turkiye

**Keywords:** Apical silica flap, Black Sea, *Craspedostauros*, epilithic marine diatom

## Abstract

*Craspedostauros* E.J. Cox is a diatom genus comprising 17 taxa reported from various regions of the world. While many species of *Craspedostauros* are epibiontic, the taxa have variable ecological preferences. In this study we formally describe *Craspedostaurosnazmii***sp. nov.**, an epilithic species discovered along the Turkish Black Sea Coast, based on light and scanning electron microscopy. *Craspedostaurosnazmii***sp. nov.** is characterized by valves that are lanceolate to narrowly lanceolate, slightly constricted near the apices with uniseriate, parallel throughout the whole valve, transapical striae and and the presence of an apical silica flap. The areolae are distributed over the valve face and the mantle. The differences and similarities between *C.nazmii***sp. nov.** and established species of *Craspedostauros* are discussed. Based on shape and morphometrics, the most similar species is *Craspedostauroscapensis*, but it is easily distinguished from *C.nazmii***sp. nov.** by its lack of an apical silica flap.

## ﻿Introduction

The genus *Craspedostauros* E.J.Cox, 1999 was established to accommodate several marine species previously included in *Stauroneis* Ehrenberg (Lange-Bertalot and Genkal 1999; Cox 1999). As the latter genus comprised mostly freshwater species, a separation was proposed by different authors to accomodate the marine species with regards to their autecology and morphological differences. Species strictly conforming with *Stauroneis* Ehrenberg are characterized by naviculoid valves with two girdle appressed chloroplasts and possess a distinct and large central area called stauros (Round et al. 1990). The valves in *Stauroneis* are flat and the girdle is composed of a few plain copulae that is always rather narrow (Krammer and Lange-Bertalot 1986; Round et al. 1990; Cox 1999).

When establishing the genus *Craspedostauros*, Cox (1999) referred to cytological (shape and number of chloroplasts) and ultrastrutural characteristics (cribrate occlusions of areolae), also leading her to point out an affinity with *Mastogloia* Thwaites ex W. Smith (1856). With regards to these affinities, *Craspedostauros* has been included in Mastogloiaceae with a recommendation to also move *Achnanthes* Bory *sensu stricto* (Cox 1999; Cox and Williams 2006) there. The molecular phylogeny published by Ashworth et al. (2017) and inferred from a 3 genes dataset (*18S*, *rbcL* and *psbC* genes) tends to associate *Craspedostauros* to *Achnanthes* and *Staurotropis*, but clearly distinguishes them from *Mastogloia* spp., strongly questionning the monophyly of the Mastogloiales. This group of genera was later referred to as CAS genera, for *Craspedostauros*-*Achnanthes*-*Staurotropis* by Mann et al. (2021), whose phylogenetic works surprisingly tended to associate the CAS genera with Bacillariaceae. However, the lack of support at the nodes or strong morphological evidences led the authors to consider this result unlikely, probably resulting from an artefact of genes/species sampling and they emphasized the need for deeper phylogenomic investigations in order to elucidate the position of the CAS genera.

As a genus, *Craspedostauros* is relatively small, with 15 taxonomically accepted species listed on AlgaeBase, to which could be added two recently described taxa from Antarctica (Trentin et al. 2022). Thus, there remains a potential for the description of new species in unexplored habitats such as tropical coasts and biofilms on seaweeds in particular (Risjani et al. 2021; Witkowski unpublished observations).

The Turkish Republic is surrounded by four different seas, namely the Eastern Mediterranean Sea, the Aegean Sea, the Marmara Sea and the Black Sea. *Craspedostaurosdecipiens* was found in the Sea of Marmara (Witkowski et al. 2000; Akçaalan and Kaleli 2021) but this is so far the only species of *Craspedostauros* discovered in Turkish Black Sea waters. The Black Sea is a semi-closed sea located in southeastern Europe. It is considered an isolated sea since the Dardanelles and Bosphorus straits limit water exchange with the Mediterranean Sea. This semi-closure influences both sea water characteristics (typically salinity) as well as the dispersal potential of aquatic species inhabiting both sides of the straits (Nevrova et al. 2013). Studying the diatoms of the Black Sea may be of additional interest for science in that they may represent flora of an ancient marine basin isolated due to limited water exchange over a fairly long geological time period (Witkowski et al. 2010).

Investigations of the diatom flora of the Black Sea go as far back as the works of Mereschkowsky (1902), whose research resulted in the description of some globally distributed diatom genera and species (e.g, *Catenula* Mereschkowsky, *Licmosphenia* and *Stauronella*). Research on the Black Sea diatom assemblages was continued by Proshkina-Lavrenko (1955, 1963) with several new species and varieties described (e.g., *Amphorainconspicua* and *Nitzschiarupestris*). Later on, Guslyakov et al. (1992) produced an atlas of benthic diatoms for the Northwestern Black Sea extensively illustrated with electron microscope images. More recently, Witkowski et al. (2010) revised *Naviculapontica* (Mereschkowsky) A.Witkowski, M.Kulikovskiy, E.Nevrova and Lange-Bertalot 2010 and *Naviculaparapontica*, A.Witkowski, M.Kulikovskiy, E.Nevrova and Lange-Bertalot 2010, whereas Witkowski et al. (2014) described *Naviculapetrovii* Nevrova, Witkowski, Kociolek and Lange-Bertalot. Nevrova et al. (2013) studied *Lyrella* and described five novel species including *Lyrellaabruptapontica*, *L.karayevae* and *L.pontieuxinii*. The Black Sea is inhabited by what seems to be an endemic taxon of the blue-pigment producing diatom, *Hasleakaradagensis* Davidovich, Gastineau and Mouget (Davidovich et al. 2012a; Gastineau et al. 2012a, 2012b). An increasing number of studies on the biodiversity and species richness of the Black Sea diatoms have been published in recent years (Nevrova 2022, 2023; Nevrova and Petrov 2019a, 2019b; Zidarova et al. 2022b). Strains of diatoms from the Black Sea have also been used to investigate either their patterns of auxosporulating (Podunay et al. 2021; Davidovich et al. 2012b, 2017a, 2017b, 2019; Kaczmarska et al. 2018), physiology (Bedoshvili et al. 2021; Davidovich et al. 2016, 2018; Podunay et al. 2016) or genomic peculiarities (Gastineau et al. 2021).

From the list above, it is obvious that it is mostly the northern part of the Black Sea whose diatom communities have been investigated and in contrast, the Southern Turkish Coasts of the Black Sea only received attention very recently (Baytut 2013). The latter authors investigated the discharge zone of the Kizilirmak River into the Black Sea and among the diatom species list many new records for Turkey were published. Similarly, Kaleli et al. (2017) studied Akliman city in the Sinop area and provided a species list also with new records for Turkish waters.

In this article, we contribute to the expanding list of novel taxa by describing *Craspedostaurosnazmii* sp. nov., a new epilithic species from the Turkish Coasts of the Black Sea. The results are based on light and Scanning Electron Microscopy. This is the first and, for now, only species of *Craspedostauros* observed along the Turkish Black Sea coast.

## ﻿Material and methods

**Figure 1.**
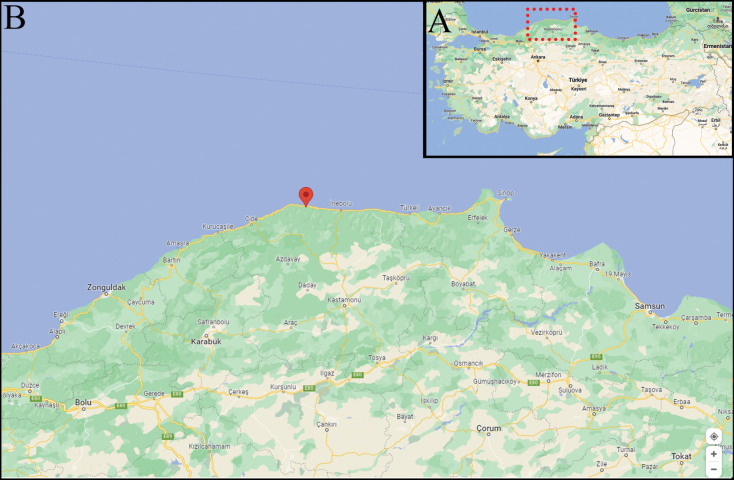
Map of the sampling location **A** shows the Kastamonu Province (red rectangle) on the shores of the Black Sea. The red dot on **B** indicates the exact place where the sampling was conducted in Doğanyurt, north of Kastamonu. Figures obtained from Google Maps, Creative Commons CCO Licence, GNU Free Document Licence.

The sample was collected in July 2017 from epilithic substrata in Kastamonu Doganyurt, Southern Black Sea (42°0'29.24"N, 33°27'34.19"E) (Fig. 1). A single epilithic sample was collected using a toothbrush from the surfaces of submerged stones of this sampling station. Environmental parameters were measured using a Lange Hach HQ40d. No live observations of the samples were conducted as the sample was processed directly and boiled with H_2_O_2_ and 10% HCl to remove organic matter and calcium carbonate respectively. After washing the diatoms with distilled water several times, permanent slides were mounted with Naphrax synthetic resin. Light Microscope (LM) observations were conducted on an OLYMPUS BX51 Light Microscope with OLYMPUS EP50 camera at Kütahya Dumlupınar University. Scanning Electron Microscope (SEM) observations were made using a FEI Versa 3D at İstanbul University with secondary electron and backscatter excitation, 10 kV and a working distance 10 mm. For this purpose, samples were placed on polycarbonate membrane filters with a 5 μm mesh. The membranes were left to dry and then attached to aluminum stubs with double-sided carbon tape, and sputter coated with ca. 20 nm gold using a Turbo–Pumped Quorum Q 150OT ES coater.

## ﻿Results

### ﻿ Craspedostauros nazmii

Taxon classificationPlantaeNaviculalesNaviculaceae

E.Yılmaz, Witkowski, Solak sp. nov.

5C996F3A-87D6-5F94-9302-AE5EEC00B59E

Figs 2 , 3

#### Type material.

**Figure 2.**
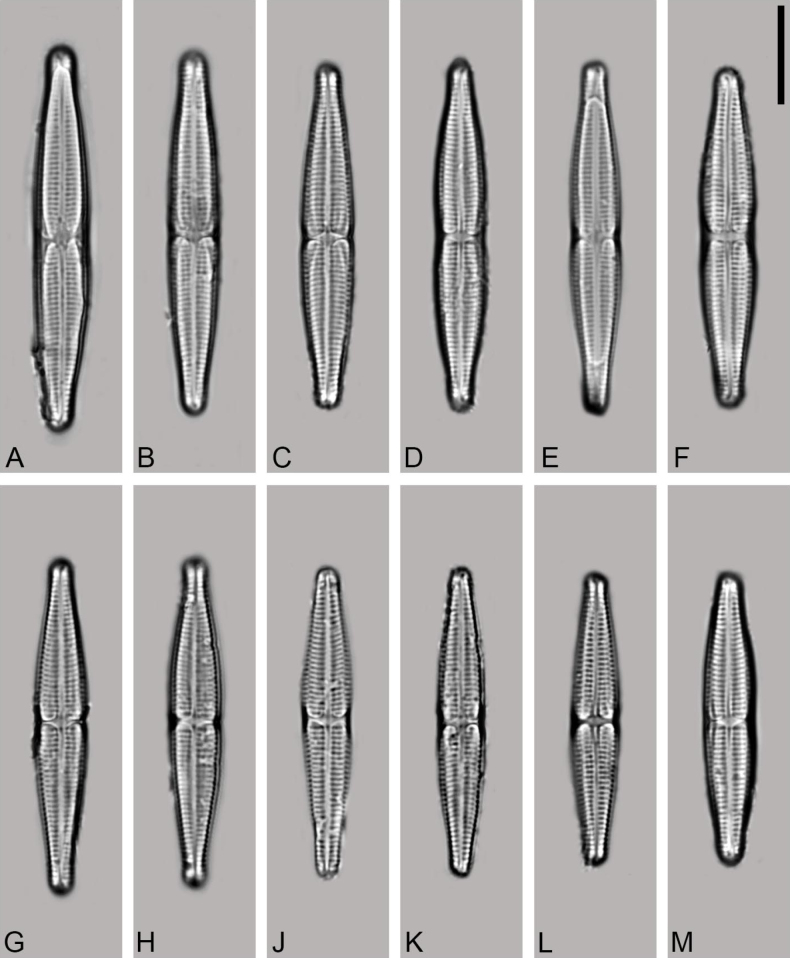
**A–M***Craspedostaurosnazmii* sp. nov., LM micrographs. Scale bar: 10 µm.

***Holotype***: Slide Number SZCZ 28843, collection of Andrzej Witkowski at the University of Szczecin. Valves representing the holotype population illustrated in Fig. 2F.

***Isotype***: Slide number TR_Kastamonu_Doganyurt_EPL_Tem2017 deposited in Kütahya Dumlupınar University (Turkey).

#### Registration.

http://phycobank.org/103900.

#### Type locality.

Turkey, Kastamonu Province, seashore in Doğanyurt District, (42°0'29.24"N, 33°27'34.19"E), collected by: Cüneyt Nadir Solak, July 18, 2017.

#### Description.

**Figure 3.**
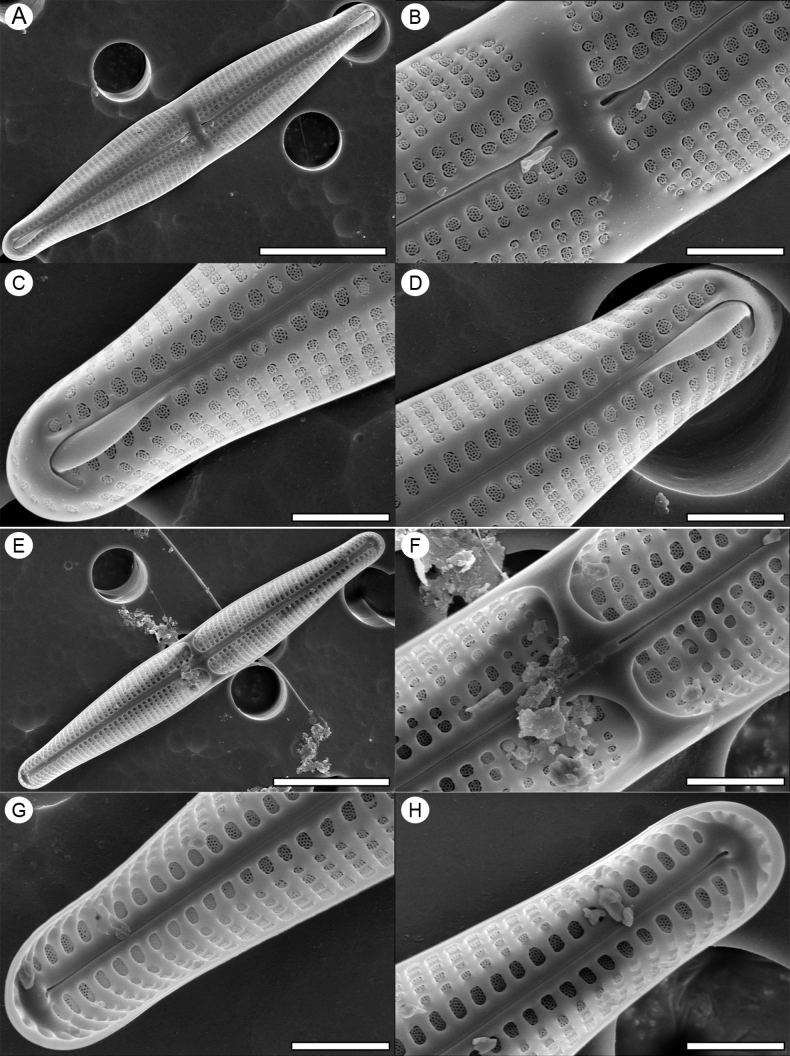
SEM micrographs of *Craspedostaurosnazmii* sp. nov. **A** external view of entire valve **B** external view of central area **C, D** external view of valve apex showing wing-like silica flap **E** internal view of entire valve **F** internal view of central area showing raphe terminate **G–H** internal view of valve apex. Scale bars: 10 μm (**A, E**); 2 μm (**B–D, F–H**).

LM (Fig. 2A–M) valves lanceolate to narrow lanceolate, slightly constricted in the middle and with rostrate to subcapitate apices, 29–42 µm in length, 4.5–5.5 µm in width (n = 50). Valves with a slight constriction in the middle, tapering towards narrowly rostrate to subcapitate apices. Axial area very narrow, but distinct, in the valve middle expanding into a central area in a form of stauros encompassing the whole valve width. Raphe branches in LM resolvable, slightly undulate, external proximal raphe endings distinct, tear-like shaped, external distal raphe endings strongly bent in same direction. Transapical striae well resolvable in LM, parallel in the middle, becoming slightly radiate and finally divergent close to apices, 20–21 in 10 µm (Figs 2A–M, 3A–D).

***SEM*** (Fig. 3A–H). Valve external view (Fig. 3A–D), valve face flat composed of one to two rows of areolae. The valve face margin marked by a distinct, continuous apically oriented siliceous rib (transformed vimineae). The transition from the valve face to valve mantle gradual in the valve middle, becoming abrupt at the apices. Hyaline area becoming larger towards to the margins in the centre. Transapical striae uniseriate, composed of 1–3 areolae on the valve face and 4–6 on the mantle in central area, and decreasing to 4 towards the apical mantle (Fig. 3B–D). The striae of the valve face in the apical part composed of a solitary areola, and increasing towards the valve middle to 2 and finally 3 near the stauros. Areolae variable in size, larger near the raphe with more pores (up to 17) in the cribrate occlusions (Fig. 3B). Raphe branches slightly undulate with external proximal ends expanded, distant from each other. External apical raphe endings strongly hook-shaped. Prominent wing-like silica flaps partially covering the first row of areolae bordering the raphe sternum present near the apices at valve secondary side (Fig. 3C–D). Valve centre with hyaline area of the stauros and symmetric with regular areolae. On one side three and on the other one to two rows of areolae at the beginning. Then, two rows of areolae on both sides and finally one row of areola towards the ends (Fig. 3A–D).

***SEM*** (Fig. 3E–H). Valve surface internally flat, narrow pore-free longitudinal lines running from apex to apex abruptly marking the face-mantle junction (Fig. 3E). Raised stauros distinctly narrower than the fascia (Fig. 3F), broadening and decreasing in thickness close to the valve margins (Fig. 3E–F). Central internal raphe fissures terminate at slight knob of silica onto rectelevatum (Fig. 3F). A flatly ended cylindrical knob present at the central nodule. Apical raphe endings terminating within prominent helictoglossae within a relatively expanded pore-free area (Fig. 3G–H). Areolae externally occluded by cribra, appearing sunken, especially close to the raphe-sternum (Fig. 3F–H).

#### Etymology.

This species is dedicated to Nazmi Yilmaz, father of the first author Elif Yilmaz in appreciation for his dedication to support and encourage her.

#### Distribution and ecology.

The species was observed in Doğanyurt District, Kastamonu Province, Black Sea. The conductivity values at the sampling station were 18.69 mS cm^-1^, DO values were 8.86 mg L^-1^, TEMP values were 15.4 °C.

## ﻿Discussion

The taxa belonging in *Craspedostauros* originate from various geographic regions of the world with *Craspedostaurosbritannicus* E.J.Cox 1999 known from the East and West coast of Great Britain, *C.neoconstrictus* E.J.Cox 1999 from the English Channel coasts, *C.australis* E.J.Cox 1999 from the south coast of Australia, *C.indubitabilis* (Lange-Bertalot and S.I.Genkal) E.J.Cox 1999 from Europe, North America and the Subantarctic Islands, *C.alyoubii* J.Sabir and Ashworth 2016 and *C.paradoxus* Ashworth and Lobban 2016 in the Red Sea and the West coast of Guam respectively, *C.amphoroides* (Grunow ex A.W.F.Schmidt) E.J.Cox 1999 in the Philippines, *C.decipiens* (Hustedt) E.J.Cox 1999 in the English Channel and North Sea coasts, *C.capensis* E.J.Cox 1999 along the West coast of South Africa and *C.laevissimus* (West and G.S.West) Sabbe 2003 in Maritime Antarctic saline lakes (Cox 1999; Sabbe et al. 2003; Rivera et al. 2011; Ashworth et al. 2017). Recently, *C.alatus* Majewska & Ashworth, 2018, *C.danayanus* Majewska & Ashworth, 2021, *C.legouvelloanus* Majewska & Bosak, 2021, and *C.macewanii* Majewska & Ashworth, 2021 were described as epibionts on sea turtles by Majewska et al. (2018, 2021). It is important to note that most of the recent discoveries originated from the Southern Hemisphere (e.g. Majewska et al. 2021; Zidarova et al. 2022a; Trentin et al. 2022).

Based on comparative morphology (Table 1), *C.alatus* in Majewska et al. (2018), *C.britannicus* E.J. Cox (1999), *C.capensis* E.J. Cox (1999), *C.indubitabilis* (Lange-Bertalot and S.I.Genkal) E.J.Cox in Rivera et al. (2011) and *C.macewanii* Majewska and Ashworth in Majewska et al. (2021) are similar taxa. Among them, *C.capensis* is the most similar taxon to *C.nazmii* sp. nov., with similar stria density and valve width. The valve outline and cribrate areolae are also similar (linear to linear-lanceolate), however the apices are not as strongly constricted as in *C.capensis*. When compared with other taxa, the stria density is higher in *C.britannicus* (~24 in 10 µm), *C.indubitabilis* (25–27 in 10 µm) and *C.alatus* (26–28 in 10 µm) than in *C.nazmii* (20–21 in 10 µm). Regarding valve outline, *C.nazmii* sp. nov. resembles the other listed taxa except *C.indubitabilis*. *Craspedostaurosindubitabilis* has a markedly elliptic outline with wider apices (Lange-Bertalot and Genkal 1999; Rivera et al. 2011: fig. 1B, C), and a larger valve width (6–7 µm). Moreover, there is no apical wing-like silica flaps (Rivera et al. 2011: fig. 1E, F). *Craspedostaurosbritannicus* and *C.alatus* also have shorter valves than *C.nazmii* (14.0–60.0, 20.0–37.0 and 29.6–41.8 µm respectively), have no apical wing-like silica flaps (Cox 1999: fig. 26) and the shape of the internal raphe endings are helictoglossae in *C.britannicus* but rectevelatum in *C.alatus*. The valve margin is straight in *C.nazmii* compared to *C.alatus* and *C.britannicus*. The average number of cribrum pores is higher in *C.nazmii* (6–17 in 10 µm) than in the other taxa (5–13 in *C.capensis*, 5(+) in *C.britannicus* and 3–11 in *C.alatus*). Unfortunately, despite numerous attempts, it was impossible to find in our samples any girdle bands that could be used for taxonomy in a similar way that was introduced by Cox (1999).

**Table 1.** Comparison of the main morphological and morphometric characters of *Craspedostaurosnazmii* sp. nov. (n = 50) with morphologically similar taxa from the literature.

	*Craspedostaurosnazmii*	*C.macewanii*	*C.capensis*	*C.britannicus*	*C.indubitabilis*	*C.alatus*
Valve outline	linear to narrow lanceolate, slightly constricted	linear to linear-lanceolate, slightly constricted	lanceolate, constricted	linear to narrow lanceolate	linear to linear-elliptic	linear to linear-lanceolate, slightly constricted
Valve length (µm)	29.6–41.8	26.0–51.0	25.0–35.0	14.0–60.0	25.0–60.0	20.0–37.0 (16.0–38.0)
Valve width (µm)	4.5–5.4	4.5–5.5	4.5–5.5	5.0–6.0	6.0–7.0	3.0–5.0 (5.0–7.0)
Stria density (in 10 µm)	20–21	28–31	19	~24	25–27	26–28(22–25)
Areolae size	variable	similar	variable	similar	similar	variable
Areolae larger along raphe side	yes	—	Yes	No	Yes	Yes
Average number of cribrum pores	6–17	highly variable	5–13	5(+)	—	3–11
Cribrum shape	rounded	rectangular-rounded	rectangular-rounded	rounded	rounded	rounded
Internal central raphe endings	slightly knob	rectevelatum + knob	knob	double helictoglossae	Knob	rectevelatum
Valve face: mantle junction	abrupt (distinct)	strong (distinct)	gradual	none	strong (distinct)	Strong (distinct)
Valve margin at centre	straight	straight	straight	slightly expanded	straight	very slightly expanded
Apical wing-like silica flaps	present	rudimentary	absent	absent	absent	present
References	this study	Majewska et al. 2021	Cox 1999	Cox 1999	Rivera et al. 2011	Majewska et al. 2018

## Supplementary Material

XML Treatment for Craspedostauros nazmii

## ﻿Additional information

### Author ORCIDs

Elif Yılmaz https://orcid.org/0000-0001-7939-1814

Andrzej Witkowski https://orcid.org/0000-0003-1714-218X

Neslihan Özdelice https://orcid.org/0000-0001-8030-7480

Cüneyt Nadir Solak https://orcid.org/0000-0003-2334-4271

Romain Gastineau https://orcid.org/0000-0001-8661-5118

Turgay Durmuş https://orcid.org/0000-0002-8242-1823

### Data availability

All of the data that support the findings of this study are available in the main text.
